# Pericranial muscle tenderness in a population based sample of chronic tension-type headache. The Akershus study of chronic headache

**DOI:** 10.1186/1129-2377-14-S1-P58

**Published:** 2013-02-21

**Authors:** K Aaseth, RB Grande, C Lundqvist, MB Russell

**Affiliations:** 1 Head and Neck Research Group, Research Centre, Akershus University Hospital, Norway

## Objective

The aims of the study were to quantify the pericranial muscle tenderness in a large population based sample of persons with chronic tension-type headache (CTTH). We also wanted to compare pericranial muscle tenderness in CTTH with the general population.

## Background

The pathofysiological mechanisms involved in CTTH are not fully understood. Several studies have shown that the tenderness of pericranial myofascial tissues are increased in patients with tension-type headache. However, little is known about CTTH characteristics in the general population.

## Methods

This is a cross-sectional population-based study. An age- and sex-stratified sample of 30,000 persons, aged 30–44 years, residing in eastern Akershus County was in 2005 drawn from the National Personal Registry. The study population received a posted questionnaire. Those with self-reported chronic headache were invited to Akershus University Hospital. All headaches were classified according to the explicit diagnostic criteria of the ICHD-II. The response rate to the questionnaire was 71%, and the rate of participation in the interview was 74%. A total tenderness score (TTS) was used to investigate pericranial tissue tenderness. 8 pairs of muscles and tendon insertions were palpated. The 4-point (0-3) scale at each location and values from left and right sides were summed to a total score. For comparison, cross-sectional data from the Danish general population using the same instruments were used.

## Results

Those with CTTH had a high TTS compared with the general population. In males, the TTS decreased significanly with age. In women, a significant relationship between headache intensity and TTS was found. Parameters like headache frequency, duration, co-occurrence of medication overuse and migraine had no significant influence on the TTS.

## Conclusions

Persons with CTTH have an increased pericranial muscle tenderness. The pathofysiological mechanisms involved in CTTH are complex and further research is needed to define the role of pericranial tissues and other factors in the genesis of CTTH.

## References

[B1] The International Classification of Headache Disorders: 2nd edition Cephalalgia 2004 14 suppl 1 1 160 AASETH K et al. Prevalence of secondary chronic headaches in a population-based sample of 30-44 year old persons. Cephalalgia 2008;28:705-13 10.1111/j.1468-2982.2003.00824.x 14979299

[B2] RASMUSSEN BK Migraine and tension-type headache in a general population: psychosocial factors Int J Epidemiol 1992 14 1138 43 10.1093/ije/21.6.1138 1483819

[B3] RASMUSSEN BK Epidemiology of headache in a general population--a prevalence study J Clin Epidemiol 1991 14 1147 57 10.1016/0895-4356(91)90147-2 1941010

